# Molecular Effects of Doxycycline Treatment on Pterygium as Revealed by Massive Transcriptome Sequencing

**DOI:** 10.1371/journal.pone.0039359

**Published:** 2012-06-19

**Authors:** Ignacio M. Larráyoz, Alberto de Luis, Oscar Rúa, Sara Velilla, Juan Cabello, Alfredo Martínez

**Affiliations:** 1 Oncology Area, Center for Biomedical Research of La Rioja (CIBIR), Logroño, Spain; 2 Ultrasequencing and Bioinformatics Core Facility, Center for Biomedical Research of La Rioja (CIBIR), Logroño, Spain; 3 Ophthalmology Service, Hospital San Pedro, Logroño, Spain; Duke University, United States of America

## Abstract

Pterygium is a lesion of the eye surface which involves cell proliferation, migration, angiogenesis, fibrosis, and extracellular matrix remodelling. Surgery is the only approved method to treat this disorder, but high recurrence rates are common. Recently, it has been shown in a mouse model that treatment with doxycycline resulted in reduction of the pterygium lesions. Here we study the mechanism(s) of action by which doxycycline achieves these results, using massive sequencing techniques. Surgically removed pterygia from 10 consecutive patients were set in short term culture and exposed to 0 (control), 50, 200, and 500 µg/ml doxycycline for 24 h, their mRNA was purified, reverse transcribed and sequenced through Illumina’s massive sequencing protocols. Acquired data were subjected to quantile normalization and analyzed using cytoscape plugin software to explore the pathways involved. False discovery rate (FDR) methods were used to identify 332 genes which modified their expression in a dose-dependent manner upon exposure to doxycycline. The more represented cellular pathways included all mitochondrial genes, the endoplasmic reticulum stress response, integrins and extracellular matrix components, and growth factors. A high correlation was obtained when comparing ultrasequencing data with qRT-PCR and ELISA results.

Doxycycline significantly modified the expression of important cellular pathways in pterygium cells, in a way which is consistent with the observed efficacy of this antibiotic to reduce pterygium lesions in a mouse model. Clinical trials are under way to demonstrate whether there is a benefit for human patients.

## Introduction

Pterygium is a very common ocular surface lesion attributed to chronic ultraviolet light exposure which typically afflicts a younger population, adding tremendous burden, both human and financial, in many countries [1]. At the cellular level, pterygium is characterized by proliferation of limbal cells, inflammatory infiltrates, fibrosis, angiogenesis, and extracellular matrix breakdown [2]–[4]. Traditionally regarded as a degenerative condition, pterygia also display tumor-like features, such as propensity to invade normal tissue and high recurrence rates following resection [5]. The mechanism of pterygium formation is not completely understood but several processes have been suggested as part of the pathogenesis of this disease, including genetic predisposition, anti-apoptotic mechanisms, cytokines, growth factors, extracellular matrix remodelling, immunological mechanisms, and viral infections [6].

Currently, the only approved treatment for pterygium is surgery [7], a group of different procedures that, in some cases, result in high recurrence rates [8]. Recently, based on the profuse irrigation of the pterygium lesion, bevacizumab-mediated antiangiogenic therapies have been tested, but these protocols have produced inconclusive results [9], [10].

In a recent article, it was shown that doxycycline, a common oral antibiotic, was able to dramatically reduce pterygium-like lesions in a mouse model [11]. The same antibiotic was responsible for reducing neovascularisation in a burn model of the rat cornea [12]. Apparently, doxycycline and other tetracyclines posses numerous properties which are independent of their antibiotic activity [13]. Specifically, the antiangiogenic effects of doxycycline seem to be related with the inhibition of matrix metalloproteinases [14], [15], although alternative and/or synergistic mechanisms cannot be excluded.

In the present study we decided to investigate the molecular effects of doxycycline on short-term primary cultures of pterygium cells, using ultrasequencing techniques, to better understand all the pathways affected by the antibiotic in pterygium.

## Results

**Figure 1:**
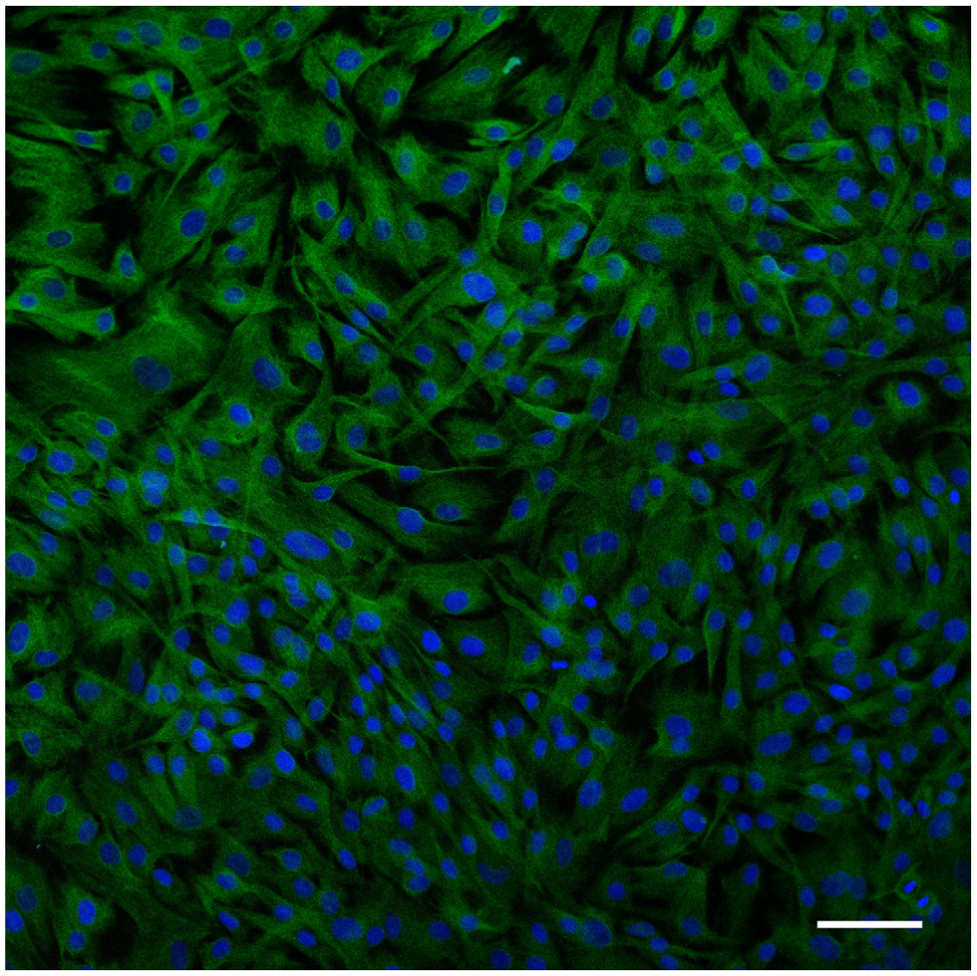
Confocal image of pterygium cells in culture stained with antibodies against vimentin (green), CD31 (white), and keratin 4 (red). DAPI was used as a nuclear counterstain (blue). All cells were vimentin-positive and no immunoreactivity was found for markers of epithelial or endothelial lineages. Bar = 20 µm.

Primary cultures were established from pterygia surgically removed from 10 patients. To identify the cell population present in these cultures, immunofluorescence for different markers was performed. Confocal microscopy of these samples showed that the predominant cell population is composed of vimentin-positive cells, with a fibroblast-compatible morphology (Fig. 1).

**Figure 2:**
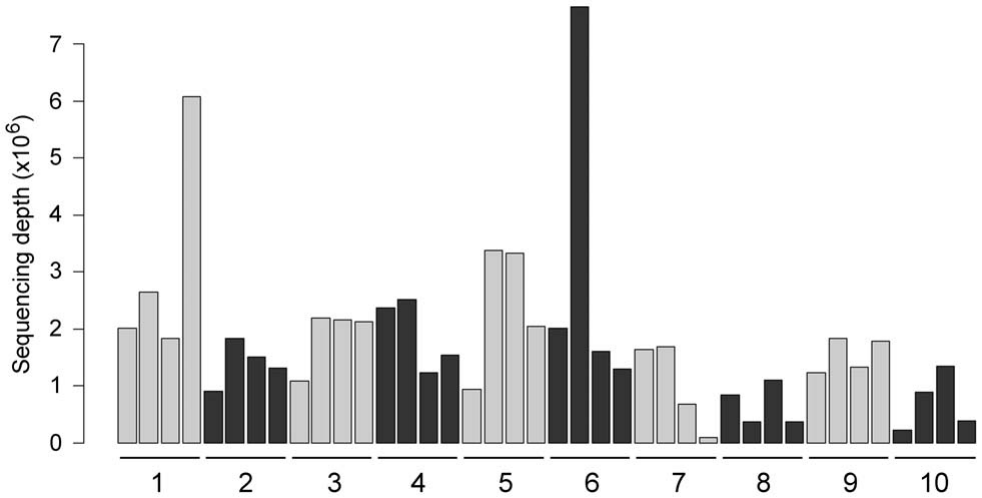
Histogram showing the number of sequences aligned for each of the 40 experimental samples (sequencing depth). Numbers represent individual donors and the 4 bars from each donor correspond to untreated control, and pterygium cells treated for 24 h with 50, 200, and 500 µg/ml doxycycline (left to right). There is a large variation in sequencing depth ranging from 82,040 reads for the less represented (cells from patient number 7, treated with 500 µg/ml doxycycline) to 7,653,010 reads for the best represented (cells from patient number 6, treated with 50 µg/ml doxycycline).

**Figure 3:**
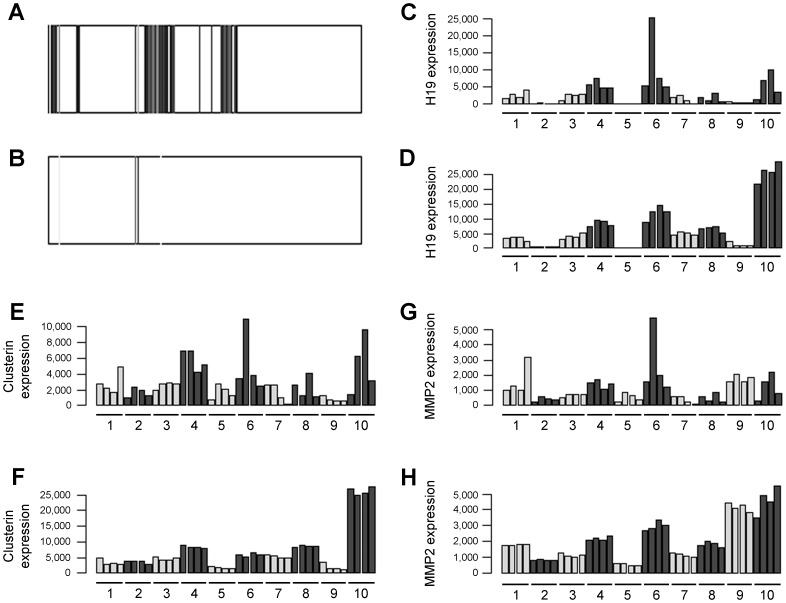
Experimental demonstration that a correct normalization protocol was applied to ultrasequencing data. Representation of the expression levels in the Y chromosome in a male (A) and a female (B) patient. Each dark line represents expression of a particular exon along the length of the chromosome. The few lines observed in females correspond to repetitive regions of the genome. Expression levels for 3 representative genes: H19 (C,D), clusterin (E,F), and matrix metalloproteinase 2 (G,H) as they appear before (C,E,G) and after (D,F,H) application of our normalization protocol. Labeling of the 40 samples is the same as in Fig.1. Normalization success can be appreciated since individual variations are larger than treatment-induced changes in these genes.

The cultures were treated with doxycycline and RNA from these samples was analyzed through massive sequencing. Due to the characteristics of the multiplexing protocol and other technical reasons, there was a broad variability on the number of sequence readings per sample, ranging from 82,040 to more than 7.5 million reads (Fig. 2). To deal with this variability, ultrasequencing data were normalized in all samples. To test the efficacy of our normalization protocol, several predictions were made and checked on the data pool. One of the tests consisted in the prediction of the patient’s gender by analyzing expression of genes coded by the Y chromosome (Fig. 3A,B) or other genes whose expression is linked to the sex of the individual, such as XIST or RPS4Y1 (not shown). The gender of all patients was correctly predicted. A second test consisted in the analysis of genes whose expression was not modified by doxycycline treatment. In these cases, the genetic background of each patient was very homogeneous in comparison with the expression in other patients, which was more variable (Fig. 3C-H).

Following normalization, the first observation was a confirmation of the predominant cell type in the cultures. Several fibroblast markers, including fibronectin 1, collagen type1 α and β, vimentin, and collagen type 3, were found among the 10 most represented transcripts of the study, whereas markers for endothelial and epithelial cells were very scarce.

**Figure 4:**
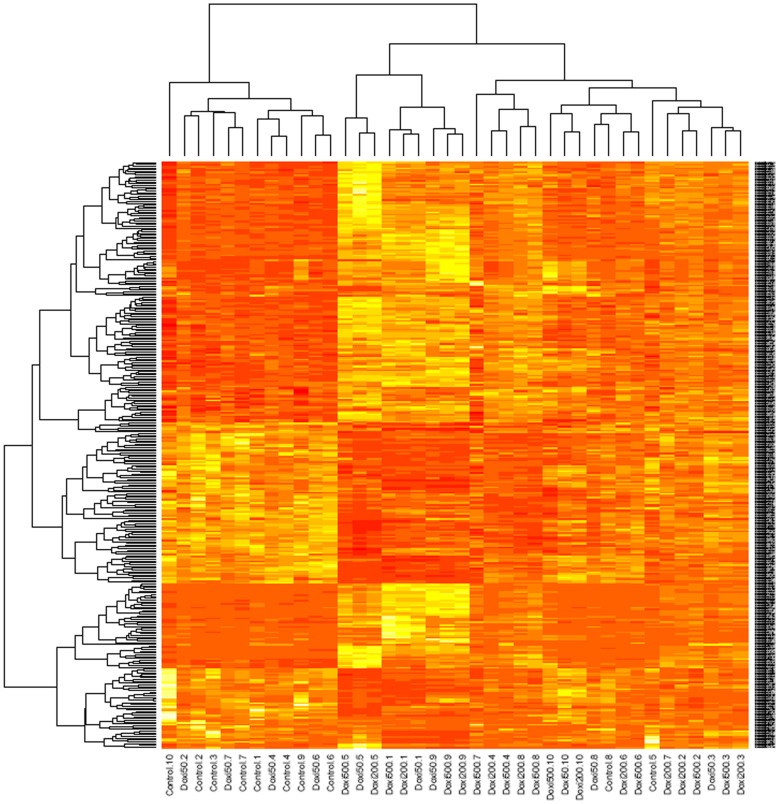
Hierarchical clustering (Manhattan plot) of all samples (columns) and the 332 genes which experienced larger changes upon treatment with doxycycline (lines). All the controls tend to cluster together (left hand side) indicating a good normalization technique. Red color expresses low expression levels and yellow designates high expression levels.

Then, gene expression of samples treated with doxycycline was compared with untreated controls. A Manhattan-type heatmap for the 332 genes whose expression experimented the largest variation after antibiotic treatment shows that treatments tend to group together, indicating that for these genes variations due to the treatment are larger than those due to individual idiosyncrasies (Fig. 4).

To investigate which cellular pathways are involved in the response of pterygium cells to doxycycline, ultrasequencing results were analyzed with specialized software. The group of genes that experiment larger variations after treatment with the antibiotic are the ones expressed by the mitochondrial genome. Eukaryotic mitochondrial DNA codes for 15 major genes; of these, a single gene (MT-ATP8) did not change, expression for the 2 genes coding for ribosomal RNA was highly upregulated in a dose-dependent manner after treatment, whereas expression for the other 12 genes was downregulated, also in a dose-dependent fashion (Table 1).

**Table 1:** Ultrasequencing values for mitochondrial genes.

Gene name	Ensembl number	Fold change		
		50 µg/mL Doxycycline	200 µg/mL Doxycycline	500 µg/mL Doxycycline
MT-ATP6	ENSG00000198899	−1.27	−1.45	−1.75
MT-CO1	ENSG00000198804	−2.04	−2.56	−2.94
MT-CO2	ENSG00000198712	−1.20	−1.56	−1.96
MT-CO3	ENSG00000198938	−1.49	−1.72	−2.38
MT-CYB	ENSG00000198727	−1.75	−2.33	−2.86
MT-ND1	ENSG00000198888	−1.67	−1.92	−2.50
MT-ND2	ENSG00000198763	−1.32	−1.39	−1.92
MT-ND3	ENSG00000198840	−1.72	−2.27	−2.70
MT-ND4	ENSG00000198886	−1.64	−1.89	−2.38
MT-ND4L	ENSG00000212907	−1.56	−2.00	−2.38
MT-ND5	ENSG00000198786	−1.47	−1.79	−2.27
MT-ND6	ENSG00000198695	−1.54	−1.92	−2.44
MTRNR1	ENSG00000211459	2.60	3.08	3.33
MTRNR2	ENSG00000210082	1.28	1.36	1.64

Values are represented as the mean fold change of 10 patients with respect to untreated controls.

**Figure 5:**
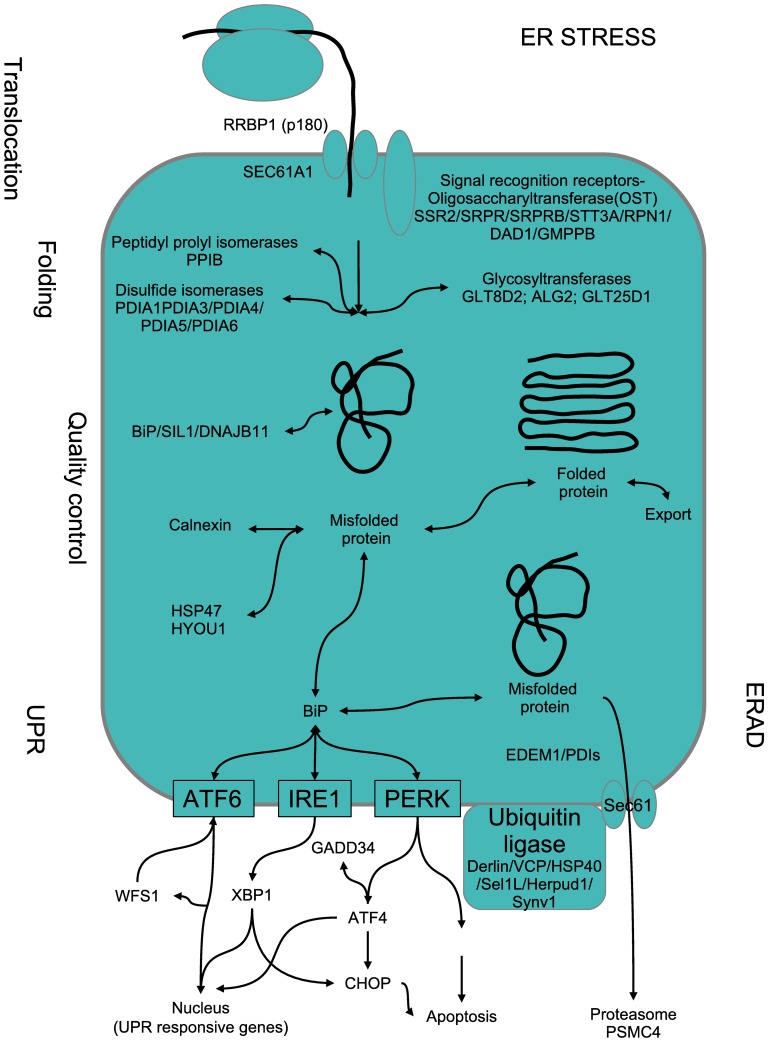
Schematic cartoon of the main pathways involved in the endoplasmic reticulum (ER) stress response. Proteins are translocated directly from the ribosome into the ER and protein folding takes place with the help of specific chaperones. If folding is correct, the proteins follow their path to the Golgi complex and the secretory pathway. If misfolded proteins accumulate in the ER lumen, the UPR can be triggered and signals will be sent to the nucleus and apoptosis could be induced through CHOP. All the proteins represented in the cartoon are significantly upregulated by doxycycline treatment.

Another pathway whose expression was deeply modified (all genes in this pathway were upregulated) upon doxycycline treatment includes almost all the proteins involved in the endoplasmic reticulum (ER) stress response (Fig. 5). This pathway is implicated at all levels, from the docking of the ribosome to the ER (SSR2, RRBP1, SEC61A1), to proteins involved in protein folding (PPIB, PDIA1 to 6, etc), quality control proteins (BiP, SIL1, HSP47), members of the unfolded protein response (ATF6, IRE1, PERK), and the ER-associated protein degradation (ERAD) process (EDEM1, SEC61, ubiquitin ligase complex, etc). When the unfolded protein response (UPR) is activated, several mediator signals are sent to the nucleus to activate UPR responsive genes and to promote apoptosis. One of these is CHOP, which is also upregulated in doxycycline treated cells.

**Table 2:** Ultrasequencing values for integrins and extracellular matrix-related genes.

Gene family	Gene name	Ensembl number	Fold change		
			50 µg/mL Doxyc.	200 µg/mL Doxyc.	500 µg/mL Doxyc.
**Integrins**	CTGF	ENSG00000118523	−2.04	−2.70	−4.55
	CRELD1	ENSG00000163703	2.60	3.40	3.47
	CYR61	ENSG00000142871	−1.82	−1.92	−2,33
	ICAM1	ENSG00000090339	2.13	3.40	3.89
	ITGA4	ENSG00000115232	−1.33	−2.04	−1.64
	ITGA5	ENSG00000161638	1.87	2.26	2.31
	ITGB1	ENSG00000150093	−1.16	−1.69	−1.35
	ITGB8	ENSG00000105855	−1.54	−2.50	−1.89
	ITGBL1	ENSG00000198542	−1.43	−1.72	−2.13
**Integrin-interacting proteins**	SLC3A2	ENSG00000168003	2.75	3.22	3.15
	THBS1	ENSG00000137801	−1.39	−1.67	−1.85
	SVEP1	ENSG00000165124	−1.44	−1.71	−2.06
**Collagens**	COL12A1	ENSG00000111799	−1.32	−1.92	−1.82
	COL5A2	ENSG00000204262	−1.25	−1.47	−1.61
**Extracellular matrix**	LAMB1	ENSG00000091136	−1.52	−2.04	−2.33
	LAMA3	ENSG00000053747	−1.47	−1.41	−1.32
	LAMA4	ENSG00000112769	−1.33	−1.82	−1.79
	SPARC	ENSG00000113140	−1.09	−1.18	−1.37

Values are represented as the mean fold change of 10 patients with respect to untreated controls.

**Figure 6:**
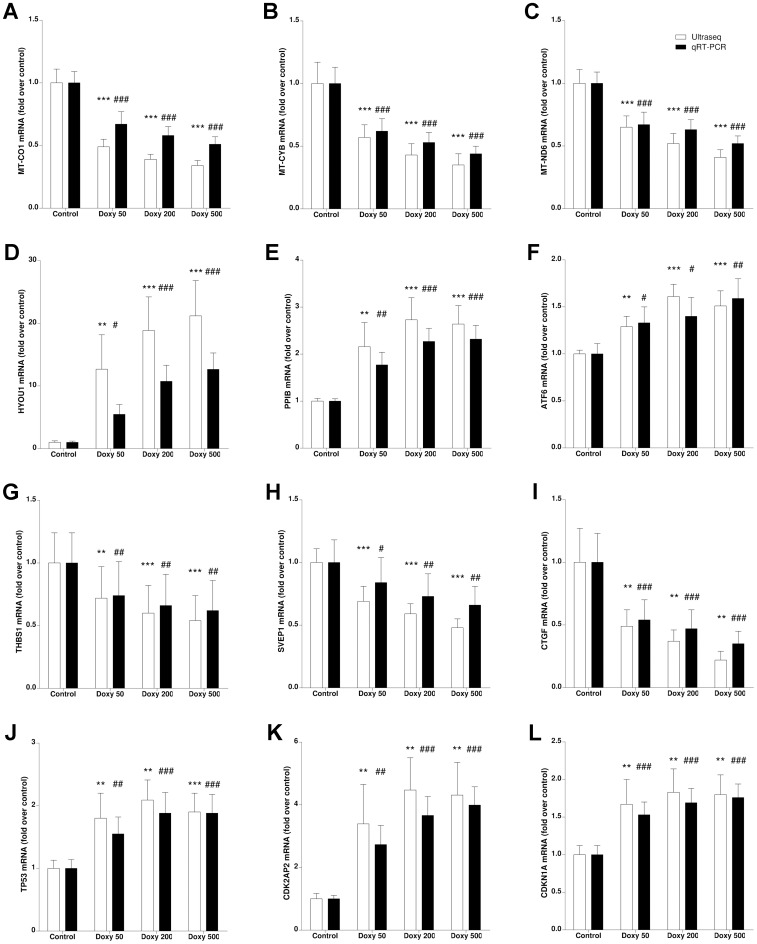
Gene expression quantification by ultrasequencing (open bars) and qRT-PCR (solid bars) for several sample genes involved in different pathways such as mitochondrial genes (A-C), endoplasmic reticulum stress response (D-F), members of the integrin family (G-I), and genes related with the cell cycle (J-L). Bars represent fold increase (or decrease) with respect to untreated controls ± SEM for 10 independent samples. Statistically significant differences with untreated controls are represented by asterisks (for ultrasequencing data) or the pound sign (for qRT-PCR). **: p<0.01; ***: p<0.001; #: p<0.05; ##: p<0.01; ###: p<0.001.

Another set of genes affected by doxycycline treatment includes several integrins, integrin-interacting proteins, collagens, and other extracellular matrix components (Table 2), indicating that doxycycline has a significant impact on matrix remodelling. In addition, several growth factors including VEGF and MANF, cytokines such as IL-6, and cell cycle related factors (TP53, CDKN1A, CDK2AP2) had also modified expression in the doxycycline treated cells.

**Figure 7:**
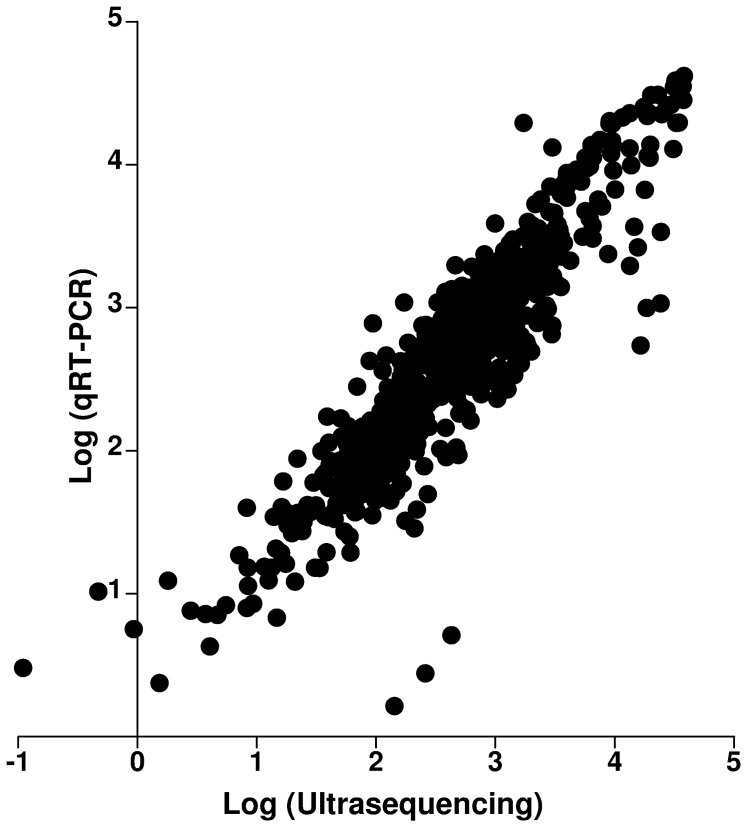
Correlation analysis between values obtained through ultrasequencing (abscises) and through qRT-PCR (ordinates). Pearsońs coefficient is 0.9110 and R^2^ = 0.8299; p<0.0001.

Sequencing results were confirmed through quantitative real time PCR (qRT-PCR) for those pathways with higher degrees of variation upon treatment. In all cases, the direction and magnitude of the change in expression was confirmed by the amplification technique (Fig. 6). For all the chosen genes, there was a tight correlation between data obtained through ultrasequencing and those collected by real time PCR (Fig. 7), indicating the efficiency of our normalization method.

**Figure 8:**
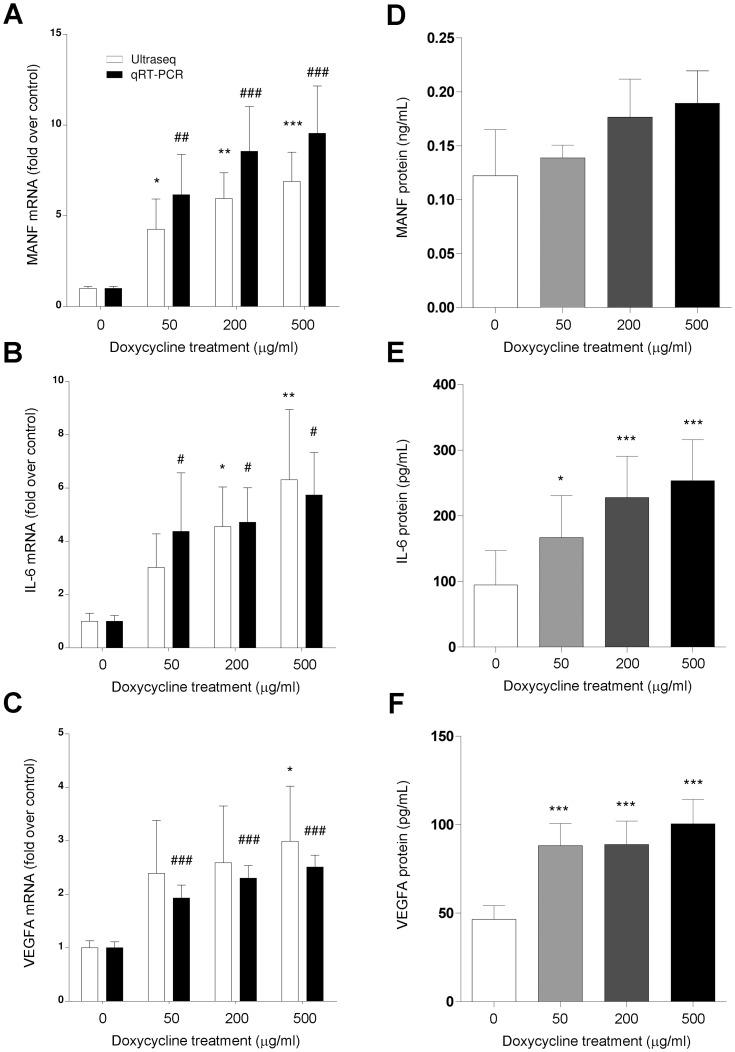
Correlation among values obtained through ultrasequencing, qRT-PCR, and ELISA for 3 secretory proteins whose expression varies with doxycycline treatment. These include MANF (A,D), IL-6 (B,E), and VEGFA (C,F). For mRNA quantification (A,B,C), all bars represent fold increase with respect to untreated controls ± SEM for 10 independent samples. For protein quantification (D,E,F), bars represent the mean ± SEM for 10 independent samples. Statistically significant differences with untreated controls are represented by asterisks (for ultrasequencing and protein data) or the pound sign (for qRT-PCR). *: p<0.05; **: p<0.01; ***: p<0.001; #: p<0.05; ##: p<0.01; ###: p<0.001.

For some genes that generate secretory proteins, such as MANF, IL-6, and VEGFA, a further confirmation was performed through ELISA protein determination in the supernatant of the treated and untreated cultures. Again, a high correlation was observed among sequencing data, qRT-PCR quantification, and ELISA protein levels (Fig. 8).

## Discussion

In this study we have shown through ultrasequencing analysis that pterygium cells respond to doxycycline treatment by modifying the expression levels of a broad number of genes responsible for important cellular functions. Among the pathways that were modified the most, we found the mitochondrial genes, the ER stress cascade, growth factors, interleukins, cell cycle regulators, integrins, and components of the extracellular matrix.

### Data Normalization

Massive transcriptome sequencing is a relatively new technique and, as such, needs some time and a series of additional developments before its complete potential can be achieved. In this regard, data normalization is one of the most relevant problems in current papers dealing with ultrasequencing data. Initially it was presumed that RNA-seq techniques were free of the nonlinear distortions that plagued hybridization techniques. Hence expression values were derived directly from the total number of alignments for a particular exon divided by its length [16]. Later on, that measurement was improved by the Reads Per Kilobase of exon model per Million mapped reads (RPKM) method [17]. Still, these and similar methods were affected by an important sequencing depth bias. Nowadays, novel normalization methods are appearing to deal with all these problems [18].

Our normalization protocol uses the broad experience accrued in the last years from microarray analysis, where quantile normalization has been the gold standard [19]. This approach does not introduce substantial biases in either genes with low expression nor in those with high expression levels. Confirmation of the quality of our approach is provided by a series of biological evidences. First, our data predicted correctly the gender of all the patients based on the expression of genes coded by the Y chromosome. Second, genes whose expression is bound to the sample genetic background were clearly differentiated with this method. In addition, expression data obtained by addition of sequencing alignments presented a high correlation with data obtained from the same samples by qRT-PCR. An outstanding advantage of this methodology is that samples with very different sequencing depth (from 80,000 to more than 7,000,000 reads, Fig. 1) can be used for the analysis whereas in other methodologies they need to be discarded in order to avoid biases [20].

### Previous High-throughput Studies in Pterygium

A few previous studies had applied high-throughput techniques to study the transcriptomics of pterygium. For instance, the gene expression of pterygium was compared to that of normal autologous conjuntiva, finding a significant increase of fibronectin, CD24, MIP-4, and NGAL in the pterygium lesions [21], [22]. Another study compared the gene expression pattern of pterygium with pre-existing data on human cornea, limbus, and conjunctiva, finding that pterygium markers closely resemble those of conjunctival and limbal cells [23]. A recent paper has shown that pterygium presents aberrant DNA methylation patterns in regions close to matrix remodelling genes such as TGM-2, MMP-2 and CD24 [24]. Our study is the first to analyze mRNA and protein changes produced by a drug treatment on pterygium cells.

Our cultured cells were positive for vimentin and negative for epithelial and endothelial markers. Therefore our first impulse was to classify them as fibroblasts. Nevertheless, Dushku and Reid showed that pterygium originates from altered limbal epithelial basal cells which express vimentin but do not stain for keratins [25], so our primary cultures seem to represent the characteristic cells of pterygium.

### Doxycycline Action on the Mitochondria

Doxycycline has been used in the past as a reversible inhibitor of mitochondrial translation [26]–[28]. Doxycycline occupies an important tRNA binding site on the 30S ribosome subunit, thus preventing the attachment of amino acyl tRNAs and terminating the translation process. Depending on the cell type, different mechanisms are activated [29]. This translation inhibition has been associated with cell arrest, apoptosis/necrosis, and cell detachment due to activation of caspase 3, 8, and 9, but also to caspase-independent mechanisms [29]–[32]. Interestingly, doxycycline concentrations achievable in serum seem to be more toxic for tumors than for normal cells [33]. To the best of our knowledge, our study provides the first evidence of doxycycline acting as a selective inhibitor of mitochondrial genes’ transcription. The inhibition is potent and raises the question of whether the recognized effect of doxycycline as an inhibitor of mitochondrial protein synthesis is due, at least in part, to the inhibition of the transcription of mitochondrial encoded genes rather than to its binding to the ribosomes’ active site.

Nonetheless, doxycycline pretreatment has been shown to counteract the apoptosis and ER stress responses induced by doxorubicin in mouse heart and testis [34], [35], probably through a preconditioning mechanism. This is in agreement with our data showing and increase in prosurvival molecules such as IL6, VEGFA, p21, or RelA. A short time exposure to doxycycline would provoke a transient mitochondrial damage and ER stress that would stimulate those survival factors that may protect the cells from the following doxorubicin insult. However in our experimental paradigm those survival factors seem to be overwhelmed by the production of proapoptotic factors such as CHOP, BAX, and caspase activation.

### Endoplasmic Reticulum Stress Response Pathway

ER stress is a cellular response to disturbances in the normal function of the ER which is conserved in all mammalian species, as well as in yeast and worms [36]. Accumulation of unfolded proteins in the ER lumen results in activation of a common response to this stress situation called the unfolded protein response (UPR), which is aimed initially at compensating for damage trying to restore normal function of the cell by halting protein translation and activating the signaling pathways that lead to increasing production of molecular chaperones involved in protein folding, but can eventually trigger cell death if ER stress is severe or prolonged [37]. There are three main signaling systems initiated by UPR through ER stress sensors, including Eukaryotic translation initiation factor 2-alpha kinase 3 (eIF2AK3), Activating transcription factor 6 (ATF-6 alpha), and Endoplasmic reticulum to nucleus signaling 1 (IRE1) [36]. In normal conditions these proteins are inactivated by Heat shock 70 kDa protein 5 (GRP78). Accumulation of unfolded proteins leads to GRP78 binding to them, resulting in release and activation of eIF2AK3, ATF-6 alpha and IRE1 [37]–[40].

Activation of eIF2AK3 induces translation of Activating transcription factor 4 (ATF-4), which is able to induce transcription of Homocysteine-inducible, endoplasmic reticulum stress-inducible, ubiquitin-like domain member 1 (HERP), DNA-damage-inducible transcript 3 (C/EBP zeta), GRP78 and Protein phosphatase 1, regulatory (inhibitor) subunit 15A (GADD34). All of them were upregulated by doxycycline treatment.

Moreover, ATF-6 has been shown to translocate to the nucleus and induce transcription of ER degradation enhancer, mannosidase alpha-like 1 (EDEM), HERP, C/EBP zeta, X-box binding protein 1 (XBP1), GRP78, and DnaJ (Hsp40) homolog, subfamily C, member 3 (DNAJC3). Again all of them were upregulated by doxycycline treatment.

In addition, active IRE1 splices XBP1 mRNA to promote translation of XBP1, which translocates to the nucleus and activates transcription of GRP78, Endoplasmin, Protein disulfide isomerase family A, member 6 (ERP5), EDEM, HERP, C/EBP zeta, Der1-like domain family, members 1, 2 and 3 (Derlin1, Derlin-2 and Derlin-3). EDEM, HERP, Derlin1, Derlin-2 and Derlin-3 promote ER-associated protein degradation, ERP5, Endoplasmin and GRP78 stimulate protein folding. Doxycycline treatment augmented the expression of all these genes.

In agreement with the results shown above, we also found an increase in the transcription of GADD34 and DNAJC3, which promote negative feed-back loops leading to restoration of protein translation. However, when the restoration of homeostasis is not achieved within a certain time lapse the UPR switches to apoptosis promotion. Despite the translational block produced by PERK activation, certain genes can bypass this block. An example is the proapoptotic protein CHOP (CCAAT/−enhancer-binding protein homologous protein) which is upregulated by ATF4 (activating transcription factor 4). CHOP causes downregulation of the anti-apoptotic mitochondrial protein Bcl-2 [41], favoring a pro-apoptotic drive at the mitochondria by proteins that cause mitochondrial damage, cytochrome c release, and caspase 3 activation. The doxycycline-mediated increase on the transcription of CHOP suggests that the proapoptotic switch has been produced. Moreover, C/EBP zeta, which is activated by IRE1 and leads to decreased Bcl-2 transcription [42], is also upregulated by doxycycline. Consistently, the expression of the pro-apoptotic molecule BAX is also increased, supporting the idea of activation of apoptosis in pterygium cells by doxycycline.

### Extracellular Matrix and Integrins

Integrins and integrin receptors regulate many aspects of cell physiology, being of particular importance in cell adhesion and migration. These two processes depend on packing of actin cytoskeleton into adhesive and protrusive organelles in response to extracellular signals. In normal epithelia, alpha/beta integrins function as receptors for the laminin family of extracellular matrix proteins (such as laminin 1 and laminin 5) and mediate the stable attachment of epithelial cells to the underlying basement membrane [43].

The function of some integrins, such as alpha-6/beta-4 integrin, is altered substantially as normal epithelia undergo malignant transformation and progress to invasive carcinoma [44]. Cooperative signaling between integrins and v-Erb-b2 erythroblastic leukemia viral oncogene homologs 2 and 3 (ErbB2 and ErbB3) is required to promote PI3K activation. This activation is mediated through GAB1 [45]. Alpha-6/beta-4 integrin can also form signaling complexes with specific growth factor receptors that act synergistically to activate PI3K [44], [46]. A signaling pathway involving EGFR, ErbB2, ErbB3, phosphatidylinositol 3-kinase (PI3K), Akt, glycogen synthase kinase 3- β (GSK3-β), and cyclin D1 is essential for the maintenance of survival, mitosis, and invasiveness of human lung adenocarcinoma cell lines as well as to evade apoptosis [47].

In our experiments, doxycycline treatment decreased the expression of multiple members involved in the interaction between the cell and the extracellular membrane, including ligands such as laminin 1, laminin 5, and THBS1, integrins such as β1 and ErbB3, intracellular mediators such as vinculin, GAB1, PI3K p85, and EIF4E3, suggesting that anoikis may be another mechanism by which doxycycline exerts its actions on pterygium cells.

### Growth Factors and Interleukins

Several growth factors such as VEGF and MANF and interleukins such as IL-6 were elevated in pterygium cells exposed to doxycycline.

VEGF has attracted much attention in the study of pterygium lesions since they are highly vascularised and upregulation of VEGF has been reported in pterygia when compared to normal conjunctiva [48]. In addition, women with a polymorphism in the VEGF gene (VEGF-460) have a 2.5-fold increased risk of developing pterygium [49]. These observations were instrumental in designing clinical trials where bevacizumab, the humanized blocking antibody against VEGF, was applied to pterygium lesions [9], [10]. The fact that doxycycline treatment results in a higher expression of VEGF suggests that this may be a compensatory reaction of the cells whose survival is compromised by the decrease in mitochondrial protein synthesis and elevated ER stress.

In the same line of thought, MANF acts as a growth factor for astrocytes and other neural cells but also as a protective factor against ER stress [50]. Since we have reported a huge increase in genes related to the ER stress response upon doxycycline treatment, MANF expression may be increased to try to compensate for these deleterious circumstances.

IL-6 is a proinflammatory cytokine which is upregulated in pterygium by UV light exposure and might be part of the pathological pathway leading to the formation of the lesion [51]. Elevation of this cytokine is related to increases in angiogenesis, cell proliferation, invasion potential, and inflammation. The upregulation of IL-6 after treatment with doxycycline may be also part of the survival program of the pterygium cells faced by the harsh conditions created by the antibiotic.

### Cell Cycle Arrest

Doxycycline has been shown to induce cell cycle arrest, although this effect is dependent on the cell line studied [29], [52]. Likewise, induction of p53 gene expression in cancer cells can lead to both cell cycle arrest and apoptosis. In addition, the expression of the proapoptotic molecule Bax is up-regulated following an increase on p53 levels [53]. In our hands, doxycycline treatment on pterygium culture increased the expression of p53 and several others CDKs (CDKN1A, CDK2AP2), as well as the proapoptotic protein Bax, providing an additional mechanism to the observed changes.

In conclusion, we have shown that doxycycline elicits a strong dose-dependent response in pterygium cells inducing a number of intracellular pathways such as mitochondrial gene expression, the ER stress pathway, genes related to the extracellular matrix environment, growth factors, interleukins, and cell cycle related proteins. These changes can explain the reduction in pterygium growth previously reported in a mouse model for this antibiotic [11] and provide the theoretical basis to pursue treatment of pterygium patients with doxycycline. Current clinical trials are under way to test whether this treatment is efficient in humans.

## Materials and Methods

### Patients and Primary Culture

Use of pterygium specimens was approved by our Institutional Review Board (Comité Ético de Investigación Clínica de La Rioja, CEICLAR). All patients provided written informed consent and the specimens were handled in accordance with the Declaration of Helsinki. Ten consecutive patients that underwent surgical removal of their primary pterygium were recruited. These included 6 males and 4 females with a medium age of 44.3±7.8 years (range 30–55). Of these, 2 patients were Spanish citizens (both women) and 8 were immigrants from South America. The excised pterygia were transferred to the laboratory for establishing cell cultures as previously described [11]. Briefly, the pterygium specimen was minced into small pieces and cultured for 3 days in DMEM/F-12 medium supplemented with 10% fetal calf serum, 200 mM L-glutamine, 0.5% DMSO, and 1% penicillin/streptomycin/amphotericin (all culture reagents are from Invitrogen, Carlsbad, CA). Once the cells had migrated from the explants and attached to the dish surface, the explant fragments were removed and the medium changed to keratinocyte-serum free medium with 5% fetal calf serum, and 1% penicillin/streptomycin/amphotericin. When the cells reached 80% confluency, they were passed to fresh dishes using 0.25% trypsin. Medium was renewed every 2–3 days and replaced with fresh medium 24 h before adding the antibiotic. All experiments were performed with cells on passages 3 to 5. For each patient, cells were subjected to 4 treatments: 0 (control), 50, 200, and 500 µg/ml doxycycline (Vibravenosa, Pfizer, Alcobendas, Spain) for 24 h.

### Immunofluorescence and Confocal Microscopy

Prior to doxycycline treatment, some cells were cultured in glass coverslips overnight, fixed with 10% buffered formalin for 10 min, permeabilized with 0.1% triton X-100 in PBS for 10 min, and exposed to a mix of 3 primary antibodies overnight at 4°C. These primary antibodies included mouse anti-vimentin (1∶40, Sigma, Madrid, Spain), rabbit anti-cytokeratin 4 (1∶100, Abcam, Cambridge, UK), and goat anti-CD31 (1∶50, Santa Cruz Biotechnology, Santa Cruz, CA). Following vigorous washes, the second layer was added for 2 h at room temperature. Conjugated antibodies in this second layer included Alexa 633 donkey anti-goat, Alexa 546 donkey anti-rabbit, and Alexa 488 donkey anti-mouse (1∶200, Invitrogen). After another series of washes, the coverslips were mounted with DAPI-containing ProLong mounting medium (Invitrogen). Paraffin sections of human uterus and appendix were processed in parallel as positive controls. A confocal microscope (Leica TCS SP5, Leica, Badalona, Spain) was used to visualize the slides.

### RNA Extraction

Total RNA was isolated from pterygium cell cultures using TRIzol (Invitrogen), purified using an RNeasy Mini kit (Qiagen, Valencia, CA), and treated with DNase I (Qiagen) following manufacturer’s instructions.

### cDNA Library Preparation and Ultrasequencing

Library preparation and ultrasequencing were performed following Illumina’s (San Diego, CA) protocols. Most reagents were also from Illumina.

First, the integrity and quality of total RNA were assessed with the Experion Automated Electrophoresis system (BioRad, Hercules, CA). Then, mRNA was isolated from 1 µg of total RNA using poly-T oligo-attached magnetic beads. This mRNA was fragmented into pieces of approximately 200 bp using divalent cations under elevated temperature. The cleaved RNA fragments were reverse transcribed into first strand cDNA using reverse transcriptase and random primers. Next, the second strand was synthesized using DNA polymerase I and RNAse H. These double-stranded cDNA fragments were end-repaired by T4 DNA polymerase and Klenow DNA polymerase, and phosphorylated by T4 polynucleotide kinase. The cDNA products were incubated with Klenow DNA polymerase to generate 3′ Adenine overhangs, therefore allowing ligation to Illumina indexing adapters to the double stranded cDNA ends. The adapter-ligated products were purified with Ampure XP magnetic beads (Agencourt Bioscience Corporation, Beverly, MA, USA) and libraries were amplified by 15 cycles of PCR with Phusion DNA polymerase (Finnzymes Reagents, Vantaa, Finland). Constructed libraries were validated and quantified using BioRad’s automated electrophoresis system Experion and qPCR respectively. Pools of 6 indexed libraries were mixed (multiplexed) at equimolar ratios to yield a total oligonucleotide mix concentration of 10 nM. Finally the resulting libraries were sequenced on the Genome Analyzer IIx platform (Illumina) to generate 150 bp single reads. Six pooled indexed libraries were sequenced in each flow cell lane.

### Data Treatment

Raw sequence data in fastq format were processed through a number of sequential steps:

1. Aggressive adapter removal: adapter sequences were removed from the raw sequences with an ad-hoc script using a non-conservative approach. Thus, even the shortest representations (one nucleotide) were eliminated.
2. mRNA alignment to DNA: alignment was performed by Tophat (an intron aware wrapper of Bowtie DNA aligner). Results were sorted and indexed using Samtools.
3. Coverage vectors for each sample were extracted under R statistical environment using Rsamtools library. Human GTF annotation from Ensembl was applied as reference for the exon genomic ranges, parsed into R data structures. Exon expression was calculated as the median coverage for every exon genomic range as stated by annotation. Exon expression data from our 40 different samples were normalized by quantile normalization and locus expression was calculated by simple addition of all the expression hits from distinct exons annotated to a single locus.
4. Samples were grouped by patient and every locus expression in the group was scaled in the interval [0,1] in order to correct for the different response ranges observed for the same gene in different genetic backgrounds.
5. Library siggenes in R was applied to select the differentially expressed loci using the FDR-based SAM method.

Resulting data were submitted to GEO (http://www.ncbi.nlm.nih.gov/geo) and are available with accession number GSE34736.

### Heatmaps

The 332 genes whose expression was most regulated by doxycycline, with a FDR lower than 0.005, were represented as a heatmap by using a Manhattan method for dissimilarity and a ward method for hierarchical clustering, calculated in an R environment.

### Pathway/Network Analysis

The Reactome FI cytoscape plugin (www.reactome.org) and the Genemania cytoscape plugin (www.genemania.org) [54] were used to identify enriched pathways and networks regulated by doxycycline treatment.

### Statistics

GraphPad Prism v5 was used for all statistics. Two-way ANOVA was used to analyze qRT-PCR and ultrasequencing data. Correlation analysis was used to compare ultrasequencing and qRT-PCR data. Differences were considered statistically significant when p<0.05.

### Confirmation of the Sequencing Results

Gene products whose expression was significantly changed upon application of doxycycline were chosen for further confirmation. cDNA was synthesized by reverse transcription of 1 µg of total RNA using the SuperScript III First-Strand Synthesis kit (Invitrogen) in a total volume of 20 µl according to the manufacturer’s instructions and was amplified by qRT-PCR using Taqman probes (Table 3) in a 7300 Real Time PCR System (Applied Biosystems, Carlsbad, CA) and their gene expression calculated using absolute quantification by interpolation into a standard curve [55]. All values were divided by the expression of the house keeping gene, GAPDH, to avoid potential pipetting errors. Some secreted proteins such as IL-6, MANF and VEGF-A were quantified in the supernatant of the cultures by commercially available ELISA kits (Uscn, Life Science, Inc. Wuhan, China).

**Table 3:** TaqMan probes used for the quantification of gene expression by qRT-PCR.

Gene family	Gene	TaqMan catalog number
ER stress	HYOU1	Hs00197328
	PDIA4	Hs01115905
	PPIB	Hs00168719
	HSPA5	Hs00946084
	ATF4	Hs00909569
	ATF6	Hs00232586
	VCP	Hs00200205
Integrins	ICAM1	Hs00164932
	THBS1	Hs00962908
	SVEP1	Hs00295944
	CTGF	Hs00170014
Cell cycle	TP53	Hs01034249
	CDK2AP2	Hs00366670
	CDKN1A	Hs00355782
Oligotransferases	SRPR	Hs01112418
	SEC61A1	Hs01037684
Mitochondria	MT-ND6	Hs02596879
	MT-CO1	Hs02596864
	MT-CYB	Hs02596867
Other	VEGFA	Hs00900055
	MANF	Hs00180640
	IL6	Hs00985639
House keeping	GAPDH	Hs99999905

Thermocycler parameters were 10 min denaturation at 95°C, followed by 40 cycles of 95°C for 15 sec and 60°C for 1 min. Values were interpolated into a standard curve to calculate absolute expression. These values were then divided by the expression of GAPDH in the same samples to correct for potential pipetting errors.

## References

[1] Coroneo M (2011). Ultraviolet radiation and the anterior eye. Eye Contact Lens 37: 214–224..

[2] Bradley JC, Yang W, Bradley RH, Reid TW, Schwab IR (2010). The science of pterygia. Br J Ophthalmol 94: 815–820..

[3] Chui J, Di GN, Wakefield D, Coroneo MT (2008). The pathogenesis of pterygium: current concepts and their therapeutic implications.. Ocul Surf.

[4] Dzunic B, Jovanovic P, Veselinovic D, Petrovic A, Stefanovic I (2010). Analysis of pathohistological characteristics of pterygium.. Bosn J Basic Med Sci.

[5] Chui J, Coroneo MT, Tat LT, Crouch R, Wakefield D (2011). Ophthalmic pterygium: a stem cell disorder with premalignant features. Am J Pathol 178: 817–827..

[6] Di GN, Chui J, Coroneo MT, Wakefield D (2004). Pathogenesis of pterygia: role of cytokines, growth factors, and matrix metalloproteinases. Prog Retin Eye Res 23: 195–228..

[7] Marcovich AL, Bahar I, Srinivasan S, Slomovic AR (2010). Surgical management of pterygium. Int Ophthalmol Clin 50: 47–61..

[8] Kandavel R, Kang JJ, Memarzadeh F, Chuck RS (2010). Comparison of pterygium recurrence rates in Hispanic and white patients after primary excision and conjunctival autograft. Cornea 29: 141–145..

[9] Mauro J, Foster CS (2009). Pterygia: pathogenesis and the role of subconjunctival bevacizumab in treatment. Semin Ophthalmol 24: 130–134..

[10] Besharati MR, Manaviat MR, Souzani A (2011). Subconjunctival bevacizumab injection in treatment of pterygium. Acta Med Iran 49: 179–183.. 18268 [pii].

[11] Cox CA, Amaral J, Salloum R, Guedez L, Reid TW (2010). Doxycycline’s effect on ocular angiogenesis: an in vivo analysis. Ophthalmology 117: 1782–1791.

[12] Dan L, Shi-long Y, Miao-li L, Yong-ping L, Hong-jie M (2008). Inhibitory effect of oral doxycycline on neovascularization in a rat corneal alkali burn model of angiogenesis. Curr Eye Res 33: 653–660..

[13] Sapadin AN, Fleischmajer R (2006). Tetracyclines: nonantibiotic properties and their clinical implications. J Am Acad Dermatol 54: 258–265.. S0190-9622(05)03231-7 [pii];10.1016/j.jaad.2005.10.004 [doi].

[14] Golub LM, Lee HM, Ryan ME, Giannobile WV, Payne J (1998). Tetracyclines inhibit connective tissue breakdown by multiple non-antimicrobial mechanisms.. Adv Dent Res.

[15] Lee CZ, Xu B, Hashimoto T, McCulloch CE, Yang GY (2004). Doxycycline suppresses cerebral matrix metalloproteinase-9 and angiogenesis induced by focal hyperstimulation of vascular endothelial growth factor in a mouse model. Stroke 35: 1715–1719..

[16] Morin R, Bainbridge M, Fejes A, Hirst M, Krzywinski M (2008). Profiling the HeLa S3 transcriptome using randomly primed cDNA and massively parallel short-read sequencing. Biotechniques 45: 81–94..

[17] Mortazavi A, Williams BA, McCue K, Schaeffer L, Wold B (2008). Mapping and quantifying mammalian transcriptomes by RNA-Seq. Nat Methods 5: 621–628..

[18] Bullard JH, Purdom E, Hansen KD, Dudoit S (2010). Evaluation of statistical methods for normalization and differential expression in mRNA-Seq experiments. BMC Bioinformatics 11: 94..

[19] Irizarry RA, Hobbs B, Collin F, Beazer-Barclay YD, Antonellis KJ (2003). Exploration, normalization, and summaries of high density oligonucleotide array probe level data. Biostatistics 4: 249–264..

[20] Tarazona S, Garcia-Alcalde F, Dopazo J, Ferrer A, Conesa A (2011). Differential expression in RNA-seq: a matter of depth. Genome Res 21: 2213–2223..

[21] John-Aryankalayil M, Dushku N, Jaworski CJ, Cox CA, Schultz G (2006). Microarray and protein analysis of human pterygium. Mol Vis 12: 55–64.. v12/a6 [pii].

[22] Tong L, Chew J, Yang H, Ang LP, Tan DT (2009). Distinct gene subsets in pterygia formation and recurrence: dissecting complex biological phenomenon using genome wide expression data. BMC Med Genomics 2: 14..

[23] Jaworski CJ, Aryankalayil-John M, Campos MM, Fariss RN, Rowsey J (2009). Expression analysis of human pterygium shows a predominance of conjunctival and limbal markers and genes associated with cell migration.. Mol Vis.

[24] Riau AK, Wong TT, Finger SN, Chaurasia SS, Hou AH (2011). Aberrant DNA methylation of matrix remodeling and cell adhesion related genes in pterygium. PLoS One 6: e14687..

[25] Dushku N, Reid TW (1994). Immunohistochemical evidence that human pterygia originate from an invasion of vimentin-expressing altered limbal epithelial basal cells.. Curr Eye Res.

[26] Ugalde C, Vogel R, Huijbens R, Van Den Heuvel B, Smeitink J (2004). Human mitochondrial complex I assembles through the combination of evolutionary conserved modules: a framework to interpret complex I deficiencies. Hum Mol Genet 13: 2461–2472..

[27] Olgun A, Akman S (2007). Mitochondrial DNA-deficient models and aging. Ann N Y Acad Sci 1100: 241–245..

[28] Pello R, Martin MA, Carelli V, Nijtmans LG, Achilli A (2008). Mitochondrial DNA background modulates the assembly kinetics of OXPHOS complexes in a cellular model of mitochondrial disease. Hum Mol Genet 17: 4001–4011..

[29] Sourdeval M, Lemaire C, Brenner C, Boisvieux-Ulrich E, Marano F (2006). Mechanisms of doxycycline-induced cytotoxicity on human bronchial epithelial cells. Front Biosci 11: 3036–3048.. 2031 [pii].

[30] Wu J, Liu T, Xie J, Xin F, Guo L (2006). Mitochondria and calpains mediate caspase-dependent apoptosis induced by doxycycline in HeLa cells. Cell Mol Life Sci 63: 949–957..

[31] Sagar J, Sales K, Taanman JW, Dijk S, Winslet M (2010). Lowering the apoptotic threshold in colorectal cancer cells by targeting mitochondria. Cancer Cell Int 10: 31..

[32] Onoda T, Ono T, Dhar DK, Yamanoi A, Nagasue N (2006). Tetracycline analogues (doxycycline and COL-3) induce caspase-dependent and -independent apoptosis in human colon cancer cells. Int J Cancer 118: 1309–1315..

[33] Rubins JB, Charboneau D, Alter MD, Bitterman PB, Kratzke RA (2001). Inhibition of mesothelioma cell growth in vitro by doxycycline. J Lab Clin Med 138: 101–106..

[34] Lai HC, Yeh YC, Ting CT, Lee WL, Lee HW (2010). Doxycycline suppresses doxorubicin-induced oxidative stress and cellular apoptosis in mouse hearts. Eur J Pharmacol 644: 176–187..

[35] Yeh YC, Lai HC, Ting CT, Lee WL, Wang LC (2007). Protection by doxycycline against doxorubicin-induced oxidative stress and apoptosis in mouse testes. Biochem Pharmacol 74: 969–980..

[36] Lai E, Teodoro T, Volchuk A (2007). Endoplasmic reticulum stress: signaling the unfolded protein response. Physiology (Bethesda) 22: 193–201..

[37] Xu C, Bailly-Maitre B, Reed JC (2005). Endoplasmic reticulum stress: cell life and death decisions. J Clin Invest 115: 2656–2664..

[38] Bertolotti A, Zhang Y, Hendershot LM, Harding HP, Ron D (2000). Dynamic interaction of BiP and ER stress transducers in the unfolded-protein response. Nat Cell Biol 2: 326–332..

[39] Patil C, Walter P (2001). Intracellular signaling from the endoplasmic reticulum to the nucleus: the unfolded protein response in yeast and mammals. Curr Opin Cell Biol 13: 349–355.. S0955-0674(00)00219-2 [pii].

[40] Hetz C, Bernasconi P, Fisher J, Lee AH, Bassik MC (2006). Proapoptotic BAX and BAK modulate the unfolded protein response by a direct interaction with IRE1alpha. Science 312: 572–576..

[41] Yoshida H, Haze K, Yanagi H, Yura T, Mori K (1998). Identification of the cis-acting endoplasmic reticulum stress response element responsible for transcriptional induction of mammalian glucose-regulated proteins. Involvement of basic leucine zipper transcription factors.. J Biol Chem.

[42] Wang XZ, Ron D (1996). Stress-induced phosphorylation and activation of the transcription factor CHOP (GADD153) by p38 MAP Kinase.. Science.

[43] Borradori L, Sonnenberg A (1999). Structure and function of hemidesmosomes: more than simple adhesion complexes. J Invest Dermatol 112: 411–418..

[44] Lipscomb EA, Mercurio AM (2005). Mobilization and activation of a signaling competent alpha6beta4integrin underlies its contribution to carcinoma progression. Cancer Metastasis Rev 24: 413–423..

[45] Shaw LM, Rabinovitz I, Wang HH, Toker A, Mercurio AM (1997). Activation of phosphoinositide 3-OH kinase by the alpha6beta4 integrin promotes carcinoma invasion. Cell 91: 949–960.. S0092-8674(00)80486-9 [pii].

[46] Bon G, Folgiero V, Di CS, Sacchi A, Falcioni R (2007). Involvement of alpha6beta4 integrin in the mechanisms that regulate breast cancer progression. Breast Cancer Res 9: 203..

[47] Sithanandam G, Smith GT, Fields JR, Fornwald LW, Anderson LM (2005). Alternate paths from epidermal growth factor receptor to Akt in malignant versus nontransformed lung epithelial cells: ErbB3 versus Gab1. Am J Respir Cell Mol Biol 33: 490–499..

[48] Jin J, Guan M, Sima J, Gao G, Zhang M (2003). Decreased pigment epithelium-derived factor and increased vascular endothelial growth factor levels in pterygia.. Cornea.

[49] Tsai YY, Chiang CC, Bau DT, Cheng YW, Lee H (2008). Vascular endothelial growth factor gene 460 polymorphism is associated with pterygium formation in female patients. Cornea 27: 476–479..

[50] Parkash V, Lindholm P, Peranen J, Kalkkinen N, Oksanen E (2009). The structure of the conserved neurotrophic factors MANF and CDNF explains why they are bifunctional. Protein Eng Des Sel 22: 233–241..

[51] Di GN, Kumar RK, Coroneo MT, Wakefield D (2002). UVB-mediated induction of interleukin-6 and -8 in pterygia and cultured human pterygium epithelial cells.. Invest Ophthalmol Vis Sci.

[52] Onoda T, Ono T, Dhar DK, Yamanoi A, Fujii T (2004). Doxycycline inhibits cell proliferation and invasive potential: combination therapy with cyclooxygenase-2 inhibitor in human colorectal cancer cells. J Lab Clin Med 143: 207–216..

[53] Lai PB, Chi TY, Chen GG (2007). Different levels of p53 induced either apoptosis or cell cycle arrest in a doxycycline-regulated hepatocellular carcinoma cell line in vitro. Apoptosis 12: 387–393..

[54] Montojo J, Zuberi K, Rodriguez H, Kazi F, Wright G (2010). GeneMANIA Cytoscape plugin: fast gene function predictions on the desktop. Bioinformatics 26: 2927–2928..

[55] Schmittgen TD, Livak KJ (2008). Analyzing real-time PCR data by the comparative C(T) method.. Nat Protoc.

